# Supplementary material for: Exceptionally high biosphere productivity at the beginning of Marine Isotopic Stage 11
Source: Nat Commun. 2020 Apr 30;11:2112. doi: 10.1038/s41467-020-15739-2 (PMC7192893; doi:10.1038/s41467-020-15739-2)
Supplement: Supplementary file 1 — Supplementary Information [file 41467_2020_15739_MOESM1_ESM.pdf]

Exceptionally high biosphere productivity at the beginning of Marine Isotopic Stage 11

Brandon* et al.

*Corresponding author: margaux.brandon@universite-paris-saclay.fr

Supplementary Information

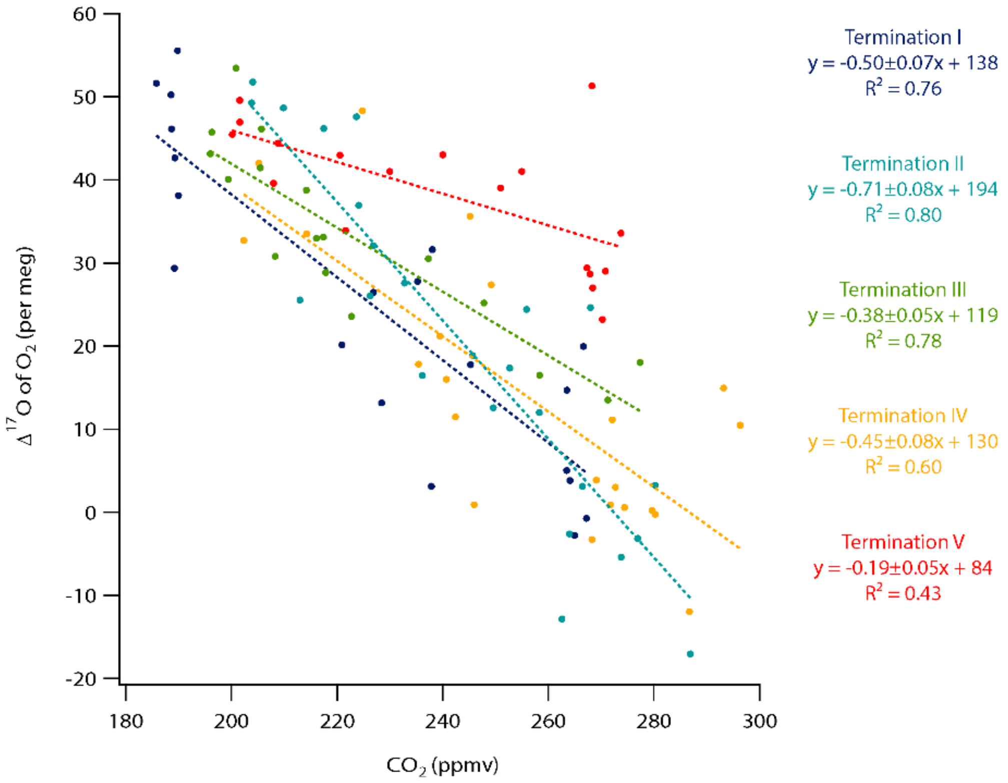

Supplementary Figure 1. Relationship between CO_2 and $\Delta^{17}O$ of O_2 for Termination I to V. The slope of the $\Delta^{17}O$ of O_2[1 and this study] vs CO_2[2] anti-correlation is detailed for each Termination. The slope over Termination V is much lower than for Terminations I-IV. The correlation coefficient is also lower for Termination V than for younger Terminations. Contrary to the younger terminations, Termination V is the only Termination when the decrease in $\Delta^{17}O$ of O_2 is not synchronous with the increase of CO_2.

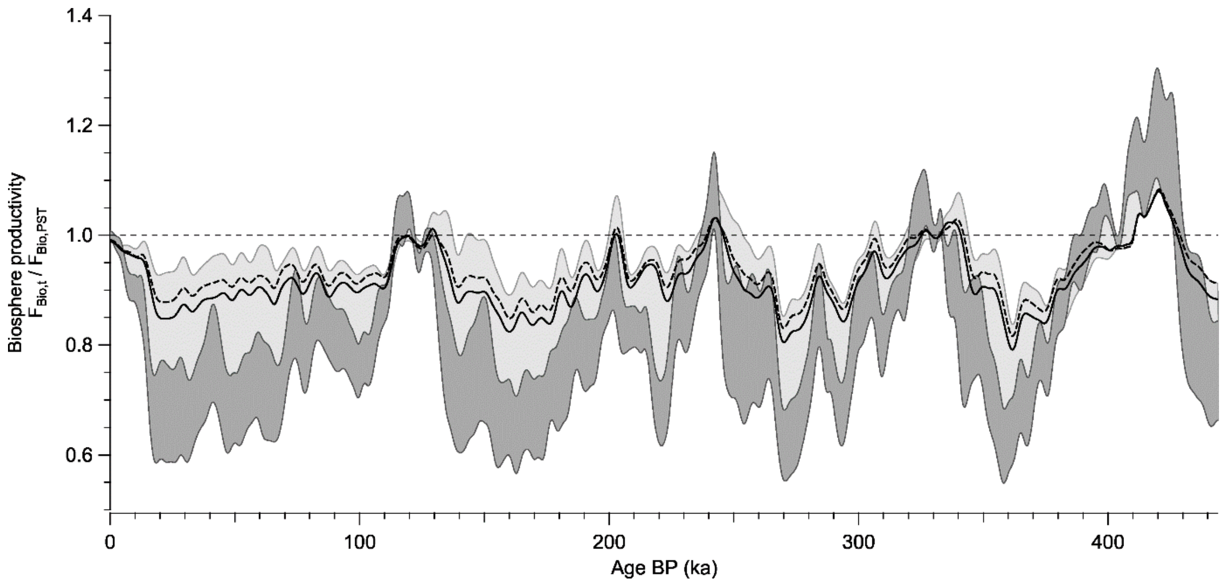

Supplementary Figure 2. Biosphere productivity reconstructions over the last 450 ka. All the lines represent the ratio of global biospheric productivity between the considered time and pre-industrial. The dark grey area represents the ratio between biosphere productivity at the considered time and pre-industrial biosphere productivity as calculated from the Landais et al. (2007) model with the uncertainty bars deduced from uncertainties in the value of $\Delta^{17}O$ of O_2 produced by the earth biosphere calculated from uncertainties in the values of the fractionation coefficients, uncertainties in the ratio of oceanic to terrestrial biosphere as well as uncertainties on isotopic composition of meteoric water linked to temporal changes in the hydrological cycle (see Method and Supplementary Table 2). The solid and dotted black lines display the ratio between biosphere productivity at the considered time and pre-industrial biosphere productivity as calculated with the model of Blunier et al., 2012[1] with two different assumptions for the isotopic composition of meteoric water: solid line is associated with a constant H_2O anomaly with time while the dotted black line was obtained with a 20 ppm lower anomaly during the glacial periods. The light grey area represents the maximum uncertainty range of the biosphere productivity reconstruction, taking into account all uncertainties listed on Supplementary Table 2 as well as uncertainty in the model used for reconstruction and the biosphere productivity reconstruction of Blunier et al., 2012[1].

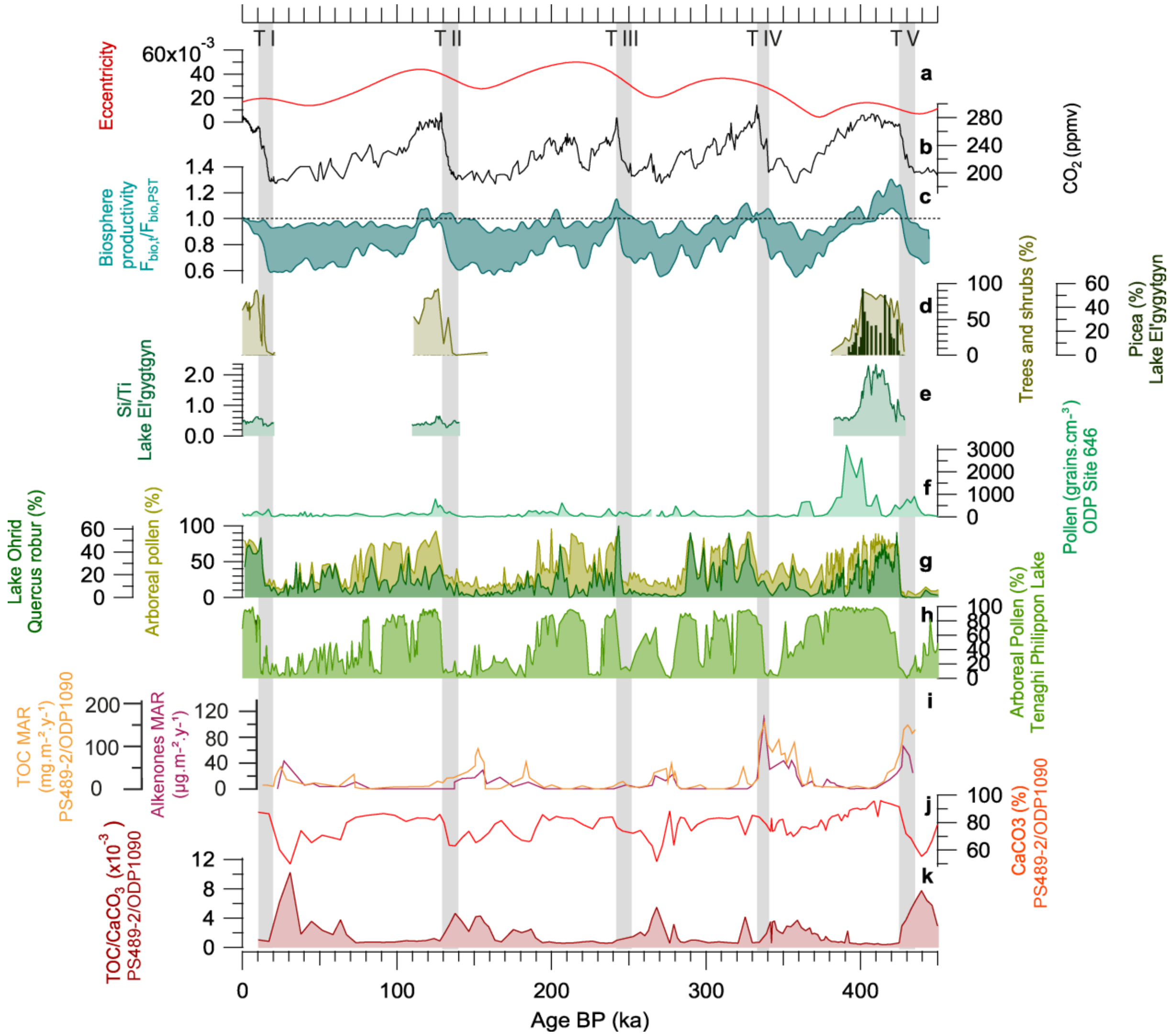

Supplementary Figure 3. Variation of the oxygen biosphere productivity compared with pollen and oceanic records. a Eccentricity[6]; **b** atmospheric CO_2[2]; **c** global oxygen biosphere productivity (this study) after interpolation to 200 years and 101 binomial smoothing with Igor software; **d** Trees, shrubs and Picea pollen record (%) from El'Gygytgyn Lake[7]; **e** Si/Ti ratio from El'Gygytgyn Lake[7], a proxy of biogenic silica normalized to detrital, reflecting the changes in diatom productivity in the lake; **f** Pollen record (grains.cm^{-3}) from oceanic core ODP 646[8] ; **g** Arboreal and Quercus robur pollen records (%) from Lake Ohrid, Balkan Peninsula[9,10]; **h** Arboreal pollen record (%) from Tenaghi Philippon Lake[11]; **i** alkenone mass accumulation rate (MAR) (µg.m^{-2}.y^{-1}) and TOC MAR (mg.m^{-2}.y^{-1}) records at Site PS2489-2/ODP1090[12]; **j** $CaCO_3$ (%) record from Site PS2489-2/ODP1090, Atlantic sector of the Southern Ocean[13]; **k** TOC/$CaCO_3$ ratio at Site PS2489-2/ODP1090, Atlantic Southern Ocean[13]. The grey shadow bars represent the period of rapid increase in CO_2 during deglaciations. Supplementary Figure

3 combines well-dated palynological and geochemical data covering the last five deglaciations and related to terrestrial and oceanic biological productivities, respectively. El'Gygytgyn core age model is based, in first order, on magnetostratigraphy and in second and third orders on the correlation between sedimentary proxy data to the LR04 stack[14] and insolation patterns[6]. The chronology of marine core ODP 646 is based on the $\delta^{18}O$ in *N. pachyderma* and on the correlation with the stack LR04 of Lisiecki and Raymo[14]. The age-model of Lake Ohrid record[9,10] is based on tephrochronology on 11 tephra layers[15] and on a second order on the tuning of biogeochemical proxy data to orbital parameters[16]. The Tenaghi Philippon core age-model is based on the correlation between vegetation changes and March and June perihelion configuration. The age-models of PS2489-2 and ODP Site 1090 were calculated using the correlation between the alkenone-based SST with the EDC ice core temperature record using EDC3 chronology[12,17,18]. Decreases in TOC and alkenone MARs from PS2489-2/ODP 1090 are correlated, indicating that TOC decrease is not a consequence of the increase in $CaCO_3$ in the sediment.

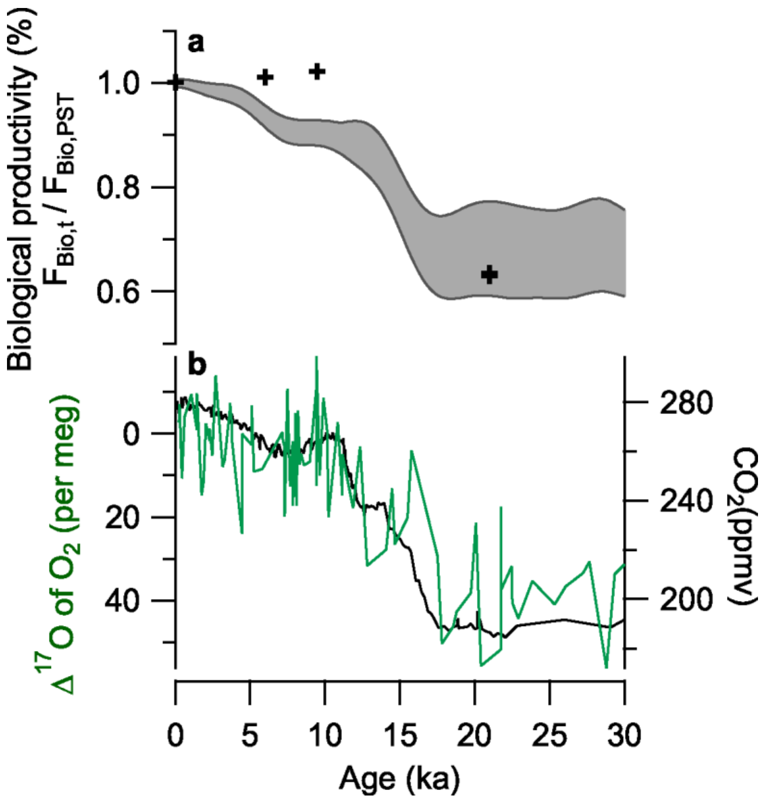

Supplementary Figure 4. Agreement between reconstructions of biospheric productivity fluxes of oxygen over the last deglaciation from $\Delta^{17}O$ of O_2 and output of coupled model equipped with vegetation and marine productivity model. a ratio between global biosphere productivity at time "t" and global biosphere productivity for pre-industrial period (expressed in O_2 flux) obtained by the coupled IPSL model equipped with the PISCES[19] and ORCHIDEE[20] models (crosses, Bopp and Kageyama, personal communication) and by interpretation of $\Delta^{17}O$ of O_2 data using the model of Landais et al. (2007)[3]and uncertainty of Supplementary Table 2 (grey envelope); **b** evolutions of CO_2 (black)[2] and $\Delta^{17}O$ of O_2 (green)[1] as in the main manuscript.

Sample	Interior sample		Exterior sample	
	$\delta O_2/Ar$	$\Delta^{17}O$ of O_2	$\delta O_2/Ar$	$\Delta^{17}O$ of O_2
**GRIP Sample 1**	-74	29	-146	13
**GRIP Sample 2**	-82	50	-134	13
**GRIP Sample 3**	-94	50	-156	26

Supplementary Table 1. Comparison of $\delta O_2/Ar$ and $\Delta^{17}O$ of O_2 values between the interior and the exterior part of the ice core

Sensitivity test	$F_{oce,PST}/F_{terr,PST}$	$F_{oce,LGM}/F_{terr,LGM}$	$F_{oce,MIS\ 11}/F_{terr,MIS\ 11}$	$\Delta^{17}O_{bio,PST}$	$\Delta^{17}O_{bio,LGM}$	$\Delta^{17}O_{bio,MIS11}$	$F_{bio,LGM}/F_{bio,PST}$	$F_{bio,MIS\ 11}/F_{bio,PST}$
Average situation	0.52	0.82	0.52	158	195	158	0.69	1.17
High $F_{oce,PST}/F_{terr,PST}$	0.59	0.82	0.52	162	195	162	0.71	1.17
Low $F_{oce,PST}/F_{terr,PST}$	0.45	0.82	0.52	153	195	153	0.67	1.18
High $F_{oce,LGM}/F_{terr,LGM}$	0.52	1.08	0.52	158	203	158	0.66	1.17
Low $F_{oce,LGM}/F_{terr,LGM}$	0.52	0.56	0.52	158	183	158	0.75	1.17
High $F_{oce,MIS\ 11}/F_{terr,MIS\ 11}$	0.52	0.82	0.59	158	195	162	0.69	1.14
Low $F_{oce,MIS\ 11}/F_{terr,MIS\ 11}$	0.52	0.82	0.45	158	195	153	0.69	1.22
Lowest estimate of the slopes associated with fractionation factor in the water cycle and biosphere (cumulative errors)	0.52	0.82	0.52	129	166	129	0.69	1.22
Highest estimate of the slopes associated with fractionation factor in the water cycle and biosphere (cumulative errors)	0.52	0.82	0.52	187	224	187	0.69	1.14
Maximum uncertainty on photosynthesis fractionation (Eisenstadt et al., 2010)[21]	0.52	0.82	0.52	193	230	193	0.69	1.14
Maximum uncertainty on the slope of respiration (Stolper et al., 2018)[22]	0.52	0.82	0.52	93	130	93	0.7	1.33
Change of hydrological cycle ($\Delta^{17}O$ of O_2 from terrestrial biosphere lower by 20 ppm during LGM and 10 ppm higher during MIS 11)	0.52	0.82	0.52	158	183	164	0.75	1.12

Supplementary Table 2. Estimates of $\Delta^{17}O_{bio}$ and associated reconstruction of the productivity for pre-industrial, Last Glacial Maximum (LGM) and MIS 11, using equations 7 and 8 with various sensitivity tests. The first 9 lines were directly taken from sensitivity tests of Landais et al. (2007) taking into account uncertainties in the ratio between oceanic and terrestrial biosphere productivity or in the estimates of the fractionation factors used for calculations of $\Delta^{17}O_{terr}$ and $\Delta^{17}O_{oce}$. Note that these fractionation factors are based on physical processes which do not vary with time so that the associated uncertainties are not independent for pre-industrial, LGM and MIS 11.

The last 3 lines correspond to 3 new sensitivity tests for the influence of fractionation during photosynthesis, possible low slope of $\delta^{17}O$ vs $\delta^{18}O$ during respiration, and possible changes in the water cycle leading to modification of the triple isotopic composition of oxygen in water (^{17}O-excess, see text)

directly transmitted to the $\Delta^{17}O$ of O_2 produced by terrestrial productivity. For all sensitivity tests, we considered extreme values so that the reconstructed global productivities are also showing extreme values.

depth	$\delta^{18}O$ (‰)	$\Delta^{17}O$ of O_2 (per meg)
EGRIP 0 m	0.0	10
EGRIP 6 m	0.19	0
EGRIP 11 m	0.16	4
EGRIP 17 m	0.08	6

Supplementary Table 3. Effect of thermal fractionation on $\Delta^{17}O$ of O_2 in the EastGRIP firn. $\delta^{18}O$ and $\Delta^{17}O$ of O_2 have been measured in four different depths in the firn of EastGRIP to evaluate the effect of thermal fractionation on $\Delta^{17}O$ of O_2. While this site registers a strong seasonal gradient, the $\Delta^{17}O$ of O_2 does not show any significant deviation from the top to 17m deep in the firn column. We can therefore conclude that there is no effect of thermal fractionation on $\Delta^{17}O$ of O_2.

Supplementary references

1. Blunier, T., Bender, M. L., Barnett, B. & von Fischer, J. C. Planetary fertility during the past 400 ka based on the triple isotope composition of O2 in trapped gases from the Vostok ice core. Climate of the Past 8, 1509–1526 (2012).

2. Siegenthaler, U. Stable Carbon Cycle-Climate Relationship During the Late Pleistocene. Science 310, 1313–1317 (2005).

3. Landais, A., Lathiere, J., Barkan, E. & Luz, B. Reconsidering the change in global biosphere productivity between the Last Glacial Maximum and present day from the triple oxygen isotopic composition of air trapped in ice cores. Global Biogeochemical Cycles 21, GB1025 (2007).

4. Eisenstadt, D., Barkan, E., Luz, B. & Kaplan, A. Enrichment of oxygen heavy isotopes during photosynthesis in phytoplankton. *Photosynthesis Research* **103**, 97–103 (2010).

5. Stolper, D. A., Fischer, W. W. & Bender, M. L. Effects of temperature and carbon source on the isotopic fractionations associated with O_2 respiration for $^{17}O/^{16}O$ and $^{18}O/^{16}O$ ratios in E. coli. *Geochimica et Cosmochimica Acta* **240**, 152–172 (2018).

6. Laskar, J. et al. A long-term numerical solution for the insolation quantities of the Earth. Astronomy & Astrophysics 428, 261–285 (2004).

7. Melles, M. et al. 2.8 Million Years of Arctic Climate Change from Lake El'gygytgyn, NE Russia. Science 337, 315–320 (2012).

8. de Vernal, A. & Hillaire-Marcel, C. Natural Variability of Greenland Climate, Vegetation, and Ice Volume During the Past Million Years. *Science* **320**, 1622–1625 (2008).

9. Sadori, L. et al. Pollen-based paleoenvironmental and paleoclimatic change at Lake Ohrid (south-eastern Europe) during the past 500 ka. Biogeosciences 13, 1423–1437 (2016).

10. Kousis, I. et al. Centennial-scale vegetation dynamics and climate variability in SE Europe during Marine Isotope Stage 11 based on a pollen record from Lake Ohrid. Quaternary Science Reviews 190, 20–38 (2018).

11. Tzedakis, P. C., Hooghiemstra, H. & Pälike, H. The last 1.35 million years at Tenaghi Philippon: revised chronostratigraphy and long-term vegetation trends. Quaternary Science Reviews 25, 3416–3430 (2006).

12. Martínez-Garcia, A. et al. Links between iron supply, marine productivity, sea surface temperature, and CO 2 over the last 1.1 Ma. Paleoceanography 24, PA1207 (2009).

13. Diekmann, B. & Kuhn, G. Sedimentary record of the mid-Pleistocene climate transition in the southeastern South Atlantic (ODP Site 1090). Palaeogeography, Palaeoclimatology, Palaeoecology 182, 241–258 (2002).

14. Lisiecki, L. E. & Raymo, M. E. A Pliocene-Pleistocene stack of 57 globally distributed benthic δ 18 O records. Paleoceanography 20, PA1003 (2005).

15. Leicher, N. et al. First tephrostratigraphic results of the DEEP site record from Lake Ohrid (Macedonia and Albania). Biogeosciences 13, 2151–2178 (2016).

16. Francke, A. et al. Sedimentological processes and environmental variability at Lake Ohrid (Macedonia, Albania) between 637 ka and the present. Biogeosciences 13, 1179–1196 (2016).

17. Jouzel, J. *et al.* Orbital and Millennial Antarctic Climate Variability over the Past 800,000 Years. *Science* **317**, 793–796 (2007).

18. Parrenin, F. *et al.* The EDC3 chronology for the EPICA Dome C ice core. *Clim. Past* 13 (2007).

19. Aumont, O., Ethé, C., Tagliabue, A., Bopp, L. & Gehlen, M. PISCES-v2: an ocean biogeochemical model for carbon and ecosystem studies. *Geoscientific Model Development* **8**, 2465–2513 (2015).

20. Krinner, G. *et al.* A dynamic global vegetation model for studies of the coupled atmosphere-biosphere system. *Global Biogeochemical Cycles* **19**, (2005).

21. Eisenstadt, D., Barkan, E., Luz, B. & Kaplan, A. Enrichment of oxygen heavy isotopes during photosynthesis in phytoplankton. *Photosynthesis Research* **103**, 97–103 (2010).

22. Stolper, D. A., Fischer, W. W. & Bender, M. L. Effects of temperature and carbon source on the isotopic fractionations associated with O_2 respiration for $^{17}O/^{16}O$ and $^{18}O/^{16}O$ ratios in E. coli. *Geochimica et Cosmochimica Acta* **240**, 152–172 (2018).
